# Modeling Trends from North American Breeding Bird Survey Data: A Spatially Explicit Approach

**DOI:** 10.1371/journal.pone.0081867

**Published:** 2013-12-13

**Authors:** Florent Bled, John Sauer, Keith Pardieck, Paul Doherty, J. Andrew Royle

**Affiliations:** 1 US Geological Survey, Patuxent Wildlife Research Center, Laurel, Maryland, United States of America; 2 Department of Fish, Wildlife, and Conservation Biology, Colorado State University, Fort Collins, Colorado, United States of America; Arizona State University, United States of America

## Abstract

Population trends, defined as interval-specific proportional changes in population size, are often used to help identify species of conservation interest. Efficient modeling of such trends depends on the consideration of the correlation of population changes with key spatial and environmental covariates. This can provide insights into causal mechanisms and allow spatially explicit summaries at scales that are of interest to management agencies. We expand the hierarchical modeling framework used in the North American Breeding Bird Survey (BBS) by developing a spatially explicit model of temporal trend using a conditional autoregressive (CAR) model. By adopting a formal spatial model for abundance, we produce spatially explicit abundance and trend estimates. Analyses based on large-scale geographic strata such as Bird Conservation Regions (BCR) can suffer from basic imbalances in spatial sampling. Our approach addresses this issue by providing an explicit weighting based on the fundamental sample allocation unit of the BBS. We applied the spatial model to three species from the BBS. Species have been chosen based upon their well-known population change patterns, which allows us to evaluate the quality of our model and the biological meaning of our estimates. We also compare our results with the ones obtained for BCRs using a nonspatial hierarchical model (Sauer and Link 2011). Globally, estimates for mean trends are consistent between the two approaches but spatial estimates provide much more precise trend estimates in regions on the edges of species ranges that were poorly estimated in non-spatial analyses. Incorporating a spatial component in the analysis not only allows us to obtain relevant and biologically meaningful estimates for population trends, but also enables us to provide a flexible framework in order to obtain trend estimates for any area.

## Introduction

Questions about the effects of global change, invasive species, disease effects, and other potential stresses on bird populations or endangered species are often focused on large spatial and temporal scales; scales at which few vertebrate data sets exist [1] [2] [3]. One such data set is the North American Breeding Bird Survey (BBS), a survey that has been conducted for more than 45 years across much of North America [4] [5]. Recently, hierarchical models for estimation of population change have been implemented within physiographic strata [6]. However, in order to provide analyses that fully reflect the extent of consequences of spatial and temporal changes that species are undergoing, we seek approaches to explicitly account for the spatial structure underlying observed patterns in abundance.

Population trends are often used to help identify species of conservation interest (e.g., [7] [8] [9] [10] [11] [12]). Several conservation organizations use population decline alone as a criterion for determining whether species need conservation attention [10]. For example, the World Conservation Union (IUCN) considers a taxon critically endangered if it declines 80% globally over a 10-year period (or three generations, whichever is longer), endangered if it declines 50–79%, and vulnerable if it declines 20–49% (http://www.iucn.org/themes/ssc/redlists/criteria.htm). Developing models that specify the associations between individuals and their habitat is essential to increase our understanding of how species use their environment [13], and for developing models that allow managers to predict the consequences of management actions. Conservation-oriented studies of spatio-temporal dynamics are especially timely as the modeling of species distribution dynamics will be useful in developing predictions about distributional changes expected to accompany climate changes, land use changes and active land management. By understanding how species’ ranges have changed over the last several decades, we can provide a basis for projections about future range changes in response to global climate change. Fortunately, a variety of hierarchical-model based approaches are now available for determining whether relationships exist between animals and environmental characteristics (i.e., to identify habitat) as well as monitor spatial or temporal changes in the populations [6] [14].

Since we are primarily interested in large spatial and long temporal scales, being able to estimate trends in occupancy and abundance across space and through time is especially relevant. Modeling trends (i.e., interval-specific proportional changes in occupancy and/or population size) while incorporating the correlation of population changes with key spatial and environmental covariates can provide insights into causal mechanisms and allow spatially explicit summaries at scales that are of interest to administrative bodies such as counties, states, parks or forest service units. We use data from the North American Breeding Bird Survey (BBS) [4] to develop spatially explicit models of temporal population change for selected species of birds to assess bird population variations and develop predictive maps for bird population trends.

Hierarchical models provide a flexible and rigorous framework for modeling BBS data [6] [15] [16]. In such models, observed BBS counts are modeled by over-dispersed Poisson counts. Over-dispersion is modeled by random effects such as year, site and observer effects. While this model accounts for site-specific dynamics, it does not account for the spatial structure underlying the abundance patterns except through post-stratification. Current BBS analyses are based on a stratification scheme defined by the intersection of states or provinces and large-scale geographic strata based on Bird Conservation Regions (i.e., BCRs). However, these can suffer from basic imbalances in spatial sampling even within a BCR because, BBS sample allocation is currently based on pseudo-random allocation *within* longitude/latitude degree blocks. As a result, for example, if we compare the density of routes between BCRs, route density is almost 3 times higher in Appalachian Mountains than in Badlands and Prairies BCRs. The density of routes is even 5.58 times higher in New-England/Mid Atlantic coast BCR than in the Badlands and Prairies BCR. Spatial imbalances in sampling effort might be observed between urban and rural areas: portions of strata located near urban centers tending to have more routes than portions located in rural areas because of observers’ availability. As a result of this, “simple averages of route data over the entire stratum would be weighted toward the parts of the stratum with the larger sample sizes, which could result in biased estimates of trends and regional relative abundances” [17]. In order to deal with this bias, post-stratification within strata should be conducted, allowing for corrected average trends accounting for this spatial imbalance. Degree blocks represent a natural unit of summary analysis and weighting for BBS data and this largely motivates the approach we adopt here. In particular, we develop a spatial model using a relatively local-scale and spatially regular stratification scheme based on the basic sample allocation unit of the BBS (degree blocks). Our model regards the degree blocks as small geographic strata, and we use a hierarchical model to define trend at the level of degree blocks, to link the degree-block level parameters using a model of spatial correlation, and then aggregate the resulting estimates to arbitrarily large post-strata such as BCRs or states. Because degree blocks constitute a natural discrete lattice, we consider the development of models based on the conditional autoregression (CAR) models. Hierarchical models, including the use of the CAR model are widely used in many small-area estimation [18] problems where sampling units (often geographic strata) contain insufficient sample sizes for estimation of stratum-specific parameters independently. Hierarchical models for small-area estimation are widely applied in many disciplines including health surveys and epidemiology [19], agriculture [20], census surveys [21] and wildlife [22].

Here we develop a spatially explicit hierarchical model for abundance trends and present analysis results for three species (Carolina wren *Thryothorus ludovicianus*, Cerulean Warbler *Dendroica cerulea* and Red-Bellied Woodpecker *Melanerpes carolinus*) to evaluate the application of such models. We also compare trend results from selected time periods for each species with trend results obtained for Bird Conservation Regions from the classical hierarchical approach currently in use for the BBS analyses [6] [15]. This approach provides a statistical framework that produces a more flexible approach to generating spatial estimates.

## Materials and Methods

### The North American Breeding Bird Survey

The BBS provides the most extensive historical database for monitoring avian populations in North America. It was initiated in the eastern United States and Canada in 1966, and expanded to provide coverage of the continental United States and southwestern Canada by 1968. Volunteer observers conduct fifty 3-minute counts along predefined roadside routes. Each route is 39.4 km long with stops at approximately 0.8-km intervals. During counts, every bird seen within a 0.4-km radius or heard is recorded. Over 5200 surveyed routes are located across the continental U.S., Alaska, and Canada. (BBS website, consulted on 12/09/2008, www.mbr-pwrc.usgs.gov/bbs/bbs.html). For each year of survey on a route, data collected include the number of stops on which individuals of a given species have been detected as well as the total number of individuals detected of the species. A variety of ancillary data such as observer names, and weather information are also collected. Those data are available online on the Breeding Bird Survey website (www.pwrc.usgs.gov/BBS).

In the non-spatial North-American Breeding Bird Survey analysis, (e.g., as presented by [6]), observed BBS counts are modeled by Poisson regression with over-dispersion. Counts for a survey route are denoted by 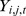 (*i* for route, *j* for unique combinations of route and observer, and *t* for year) and are independent Poisson random variables with means 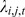 that are log-linear functions of explanatory variables,
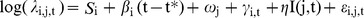



Explanatory variables are stratum-specific intercepts (*S*) and slopes (*β*, t* is the baseline year), observer/route combinations (*ω*), year (*γ*), start-up (*η*, with *I(j,t)* an indicator that takes the value 1 for an observer’s first year of survey on a route, 0 otherwise), and overdispersion effects (ε) [6]. Strata in Sauer and Link (2011) [6] are physiographic-region based on BCRs within states or provinces.Using such a model, the estimation of stratum abundance data *n_i,t_* for stratum *i* and year *t* is defined in terms of model components and their variances:
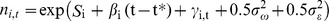



Here, trends can easily be estimated as a change in abundance between two periods of time. While alternative statistical definitions of trend exist (e.g. [23]), the current way of estimating trends in the BBS analyses is done using the definition proposed by Link and Sauer [15] [24]. Trend is defined as an interval-specific geometric mean of yearly changes in population size, expressed as a percentage. The trend from year 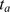 to year 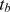 for cell *i* is 100(B_i_−1)%, where:
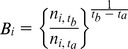



### Conditional Autoregressive Model

We overlay a grid on the BBS survey area and we estimate the trend for a theoretical route contained within each individual grid cell. Grid resolution can be adapted to each species-specific range, but our analyses use a 1-degree block grid because this represents the basic sample allocation unit of the BBS. Each route that has a starting point that falls into a cell is considered to belong to that cell. Using a simple grid that is applied on the area of interest, we can i) shift from the set of BBS routes with an irregular geographical repartition to a lattice process; ii) divide the entire BBS sampling area into patches of equal area; and finally iii) easily define the underlying spatial structure. A connection matrix based on distance (using distance between cells’ centroids) or neighboring relationship can be defined to specify relationships among cells. This approach facilitates computation: the same statistical model can be applied at different scales simply by changing the resolution of the grid (i.e. cell size). Analyses presented here use a 1-degree block grid, allowing for fast computation and providing good preliminary results. The spatial effect is modeled via a Gaussian CAR model [25]. This type of model can be thought of as spatial analogs of autoregression models in time-series, in the sense that the model is described by relating values of the state process to neighboring values (in the case of time-series, preceding values). Therefore we model the observed count *Y_i,t_* on a route *i* at year *t* by a Poisson distribution with mean *λ_i,t_*, which depends on a year-specific intercept, an observer effect, and a spatial effect at the level of the cell encompassing the corresponding route:
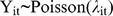
with
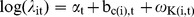
where α*_t_* is the year-specific intercept, and *ω_K(i,t)_* is the observer effect for the observer *K* on the route *i* during year *t*. Since observed patterns of change in counts do not only reflect changes in population sizes, but also changes in the pool of observers (or other factors affecting detection), it is necessary to incorporate this observer effect in the modeling process. The parameter *b_c(i),t_* is the spatial effect for the cell *c* that encompasses the route *i*. We decided to allow the spatial structure to be different for each year, hence the year index on *b*.

We used diffuse normal distributions (a customary vague prior) for the year effect parameters
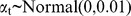



The observer effects were assumed to be normal random variables with mean 0 and variance *σ^2^*:
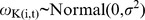
with
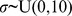



The spatially correlated random effect *b_c(i),t_* is expressed as a CAR model where the spatial effect of the cell *c* is based on the grid cells that share a common boundary with cell *c*. Specifically, we use an intrinsic version of the CAR model analogous to that proposed by Besag *et* *al.* (1991) [25]. The Gaussian CAR model can then be defined aswhere *M* is a *C*x*C* diagonal matrix with elements *M_cc_* proportional to the conditional variance of *b_c,t_*|*B_−c,t_* and 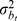 is the conditional variance parameter. In the intrinsic model, we set *M_c_* = 1/*n_c_*. Essentially, *b_c,t_* has a normal distribution with conditional mean given by the average of its neighbors. We can note that the conditional variance is inversely proportional to the number of neighbors of *b_c,t_*. We define the *C*x*C* symmetric matrix of the neighborhood weights *W*. The element *w_ck_* illustrates the connectivity between cell *c* and cell *k*. In our case, we set *w_ck_* = 1 if cells *c* and *k* are adjacent, and *w_ck_* = 0 otherwise (we also set *w_cc_* = 0). Row sum is given by 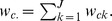 Since the grid structure is time invariant, and therefore spatial arrangement of the cells does not change over time, weights are constant over time. Let *B_t_* be the vector [*b_1,t_*…., *b_C,t_*], and *B_−c,t_* the corresponding vector that omits *b_c,t_*.

Thogmartin *et* *al.* (2004) [26] have previously developed a spatial model for BBS population change that included a CAR model to account for spatial autocorrelation that occurs between survey routes. However, in the model they used, the spatial autocorrelation is constant over time, a more constrained model, and one that does not accommodate spatial variation in trend. Moreover, they based their spatial structure on a network of routes by delineating a spatial neighborhood on an irregular lattice by tessellating the sample routes. This makes the spatial effect strongly dependent on the realized sample locations, whereas a lattice grid would provide estimates independent of the routes and their repartition. Moreover by using a degree-block grid, several routes may fall in one cell, leading to estimates reflecting the quality and basic design of the sampling scheme. Here, we focused on developing a statistical model that can easily be used as a reporting tool and therefore had to be operational and applicable to all species every year, and in which covariables (such as climate covariates) could be easily added. This practical efficiency is one key element of this work, since one of the ultimate goals is to implement this approach as a web-based tool, possibly through a R-package that would allow managers to conduct their own analysis.

### Trend

Following the current approach in the BBS analysis, trend is defined as an interval-specific geometric mean of yearly changes in population size, expressed as a percentage. Therefore, the trend 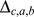 from year 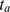 to year 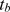 for cell *c* is still defined as:
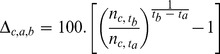
where *n_c,t_* would represent the expected abundance at time *t* of a theoretical route in cell *c* and is specified as:
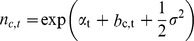



We used a Bayesian analysis (e.g. [6]) to obtain posterior samples of *n_c,t_* for each cell and each year, from which we computed the trends of interest.

### Bird Conservation Regions Post-stratification

Having parameterized the model based on a uniform grid, obtaining trend estimates for any geographical region in the area of interest is straightforward, as long as we know the shape of this region. To illustrate and compare our results with those of current BBS analyses, we obtained estimates of population trends for the Bird Conservation Regions (BCR). A map of the different BCRs can be found online at www.nabci-us.org/map.html.

BCR trends are defined as a weighted average of estimates of cells belonging to a given BCR depending on how much area of each cell of those cells is part of the BCR of interest. To estimate BCR-level trends, we computed for each cell the proportion of the cell area contained in the BCR and used this as a weight. Then, we used this weight value in the weighted average of all the cell estimates to determine the global trend estimates for each BCR.

Therefore, if we have a set of *K* BCR regions {*1,2,…k, …,K*}, and of *C* cells {*1,…,c,…C*}, the general equation to compute the trend for BCR *k* between year *a* and *b* will be:
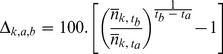
with
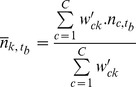
and
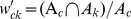
where *A_c_* and *A_k_* are the area of the cell *c* and of the BCR *k* respectively.

Moreover, since Earth is not flat but approximately spherical, and we are using degree blocks of latitude and longitude as our cells, actual cell area will vary with latitude, and we need to consider this when averaging theoretical routes representing each cell. Therefore, when computing BCRs’ trend estimates, we need to correct the weight of each cell that belongs to this BCR by the relative area of this cell compared to the other cells in the BCR. If we have two cells *n* and *s* that both covered half-way by a BCR *r*, the weights *w'_nr_* and *w'_sr_* are both equal to 0.5. However if cell *n* is close to the North Pole, and cell *s* is closer to Equator, cell *s* is larger than cell *n* and therefore contributes more to the BCR *r*. So, its weight in the computation of the BCR trend should be more important. To achieve this goal, we simply divide the area of each cell by the mean area of cells contained in the BCR of interest. Then, 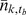 becomes:
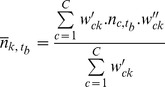
with
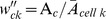
where 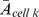 is the average cell area in BCR *k*. We note that it would be straightforward to adopt a model grid based on equal area units using a standard map projection. However, our motivation for using the degree-block model grid is that it is the sample allocation unit of the BBS.

If we take *R* as the radius of Earth, it is straightforward to show that the area of a cell *c* which has for upper left corner coordinate (long1, lat1) and for lower right coordinates (long2,lat2) can simply be expressed as:
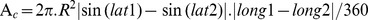



For our comparison, we have decided to focus on population trends for each species between 1999 and 2009. Results are shown in table 1.

**Table 1:** Trends in United States Bird Conservation Regions (BCR) between 1999 and 2009: comparison between results obtained with the Link & Sauer (2002) hierarchical model for population change and our conditional autoregressive (CAR) model.

BCR	Carolina Wren				Cerulean Warbler				Red Bellied Woodpecker			
	CAR		Link & Sauer		CAR		Link & Sauer		CAR		Link & Sauer	
*Appalachian Mountains*	1.9	[0.5; 3.2]	2.2	[1.0; 3.45]	−2.8	[−7.9; 2.4]	−1.0	[−3.1; 1.7]	3.4	[2.1; 4.7]	3.8	[2.9; 4.6]
*Atlantic Northern Forest*	**8.6**	**[0.2; 17.7]**	**11.6**	**[**−**10.7; 33.4]**	–			–	**11**	**[5.3; 17]**	**19.7**	**[7.4; 30.5]**
*Boreal Hardwood Transition*	–			–	**6.6**	**[**−**12.3; 29.1]**	−**17.5**	**[**−**62.0; 10.7]**	**6.7**	**[1.5; 12.1]**	**9.7**	**[3.6; 15.5]**
*Central Hardwoods*	2.1	[0.7; 3.5]	2.6	[1.4; 3.9]	3.8	[−5.3; 13.3]	−2.1	[−4.7; 0.9]	0.1	[−1.1; 1.4]	−0.04	[−1.0; 0.9]
*Central Mixed Grass Prairie*	3.1	[−1.9; 8.2]	3.1	[−2.0; 8.1]	–			–	1.8	[−1.5; 5]	2.8	[1.1; 4.7]
*Eastern Tallgrass Prairie*	4.7	[2.3; 7.1]	5.5	[3.2; 7.8]	**0.1**	**[**−**10; 11.5]**	−**3.8**	**[**−**15.4; 13.5]**	2.9	[1.4; 4.3]	2.0	[0.9; 3.1]
*Edwards Plateau*	1.3	[−4.4; 6.3]	0.5	[−3.5; 4.0]	–			–	−**2.7**	**[**−**9; 3.7]**	**0.3**	**[**−**12.6; 14.6]**
*Gulf Coastal Prairie*	1.1	[−2.7; 5.1]	5.8	[3.0; 9.4]	–			–	−1	[−4.4; 2.7]	2.3	[0.1; 4.5]
*Lower Great Lakes/St. Lawrence Plain*	**2.4**	**[**−**2.1; 7.1]**	**3.1**	**[**−**2.1; 8.3]**	−8.3	[−18; 3.5]	−1.6	[−6.4; 3.0]	6.4	[3.5; 9.4]	6.7	[5.0; 8.5]
*Mississippi Alluvial Valley*	1.3	[−0.4; 2.9]	1.3	[−0.2; 2.7]	–			–	−3.1	[−4.8; −1.4]	1.2	[−0.5; 2.7]
*New England/Mid-Atlantic Coast*	5.3	[3.8; 6.8]	4.7	[3.2; 6.4]	−**7.9**	**[**−**27.2; 15.6]**	**3.6**	**[**−**24.4; 21.1]**	5	[3; 7.1]	4.1	[2.7; 5.6]
*Oaks And Prairies*	−0.3	[−2.5; 1.9]	−0.7	[−2.7; 1.2]	–			–	4.8	[2.4; 7.4]	2.6	[0.8; 4.6]
*Peninsular Florida*	0.2	[−1.5; 1.9]	−0.2	[−1.2; 0.7]	–			–	−0.9	[−2.5; 0.7]	−0.7	[−2.0; 0.6]
*Piedmont*	1.3	[0; 2.6]	0.8	[−0.3; 2.0]	−3.6	[−15.3; 9.5]	−0.3	[−7.7; 7.7]	1.6	[0.2; 3.1]	1.4	[0.4; 2.4]
*Prairie Hardwood Transition*	**5.3**	**[**−**2.1; 13.2]**	**5.1**	**[**−**6.2; 13.9]**	6.7	[−6.2; 21.7]	−1.1	[−5.4; 7.3]	5.4	[3.6; 7.3]	4.9	[3.8; 6.2]
*Prairie Potholes*	–			–	–			–	3.8	[−2.6; 10.5]	5.1	[1.4; 9.1]
*Southeastern Coastal Plain*	0.6	[−0.4; 1.6]	0.5	[−0.2; 1.1]	**6.7**	**[**−**14.8; 32.1]**	−**3.0**	**[**−**53.0; 105.1]**	1.6	[0.6; 2.5]	1.0	[0.5; 1.5]
*Tamaulipan Brushlands*	−6.9	[−18.1; 4.4]	6.9	[1.3; 14.0]	–			–	–			–
*West Gulf Coastal Plain/Ouachitas*	0.5	[−0.8; 1.8]	0.4	[−0.9; 1.6]	7.8	[−26.3; 56.2]	−15.3	[−27.6; −0.4]	1	[−0.7; 2.7]	0.7	[−0.3; 1.6]

Results indicating a greater precision thanks to the spatial approach are indicated in bold.

### Implementation in WinBUGS

We implemented this model and conducted the analysis for each species using the free software WinBUGS 1.4.3 [27] called from R 2.12.0 [28] using the R library R2WinBUGS [29]. For each of the three species discussed below, we ran 3 chains using non informative priors and based our inference on 20,000 samples from the posterior distribution of parameters, after 5,000 discarded iterations. The WinBUGS code is provided in Appendix S1, and includes area computations described above. Convergence was checked using the Rhat statistics obtained from R2WinBUGS, and from the Gelman-Rubin Diagnotics available in WinBUGS.

### Case Studies: the Carolina Wren, the Cerulean Warbler and the Red-bellied Woodpecker

We present here three different case studies: the Carolina wren, the cerulean warbler and the red-bellied woodpecker to illustrate the application of including the spatial component in a trend analysis model. These three species were chosen because their population dynamics are well-known which should allow us to verify if our model provides precise and biologically meaningful trend estimates. We decided to use climate data to pick the most biologically relevant intervals for each species in choosing the appropriate time intervals (i.e., the year between which the trend must be estimated),. Relevant parameters considered include the presence of severe winters (with temperature under the average of winter temperatures), high and low precipitations, and/or periods of global increase or decrease in temperature.

The Carolina wren is a non-migratory species, present in a wide range of habitats (from swamps to forest and residential area) in the eastern United States and around the Gulf of Mexico [30]. It is especially sensitive to cold weather leading to frequent drops in population following severe winters, although populations increase rapidly in years with mild winters [31]. While this species is not considered to be of conservation concern, the Carolina wren provides a useful test case for our modeling purposes because of its relatively reduced range, its large population fluctuations that vary spatially within this range, and its recognizable and loud song that make it easily observable along BBS routes. We expect this species to undergo extreme fluctuations in portions of their ranges following severe winters when snow cover may prevent them from foraging effectively on the ground. Therefore, trends for the Carolina wren were computed from 1966 to 1976, from 1979 to 1983, from 1984 to 1989, and from 1990 to 1999, all time periods punctuated with severe winter weather events.

The cerulean warbler is a migratory species, and is a specialist of the upper canopy of extensive mature deciduous woods. It breeds in the upper Ohio River Valley and Allegheny region, and its range has expanded into the northeastern USA. Declines in its breeding range (mainly in the Midwest) have been observed [15]. These reductions are thought to be caused by destruction of overwintering habitat in northern South America [32]. Facing continuing habitat loss and fragmentation on its breeding and wintering areas, the cerulean warbler population has globally undergone a rapid decline and as such has been assigned a vulnerable status by the IUCN [15] [33]. Because of the generally consistent but regionally varying declines [15], and the variation in abundance in the breeding population area, cerulean warblers are a good model species. We know that this species’ population is declining, and that rates of decline vary by region. Therefore, we expect the trend patterns to be spatially less smooth than for the Carolina wren or the red-bellied woodpecker. We estimated trends for the cerulean warbler between 1966 and 2001, between 1966 and 1975, between 1977 and 1987, and finally between 1990 and 1999. Since this species is migratory and should not be affected by winter conditions, time intervals were chosen to provide trends both over a long time-period (1966–2001), and for shorter regular intervals not directly related to specific climatic events.

The red-bellied woodpecker is considered a sedentary species, and is widely distributed throughout the eastern half of the United States, ranging from the western wooded portions of the Great Plains(for its western limit range) to the south of Ontario (for its northern limit range) [34]. Breeding records from Connecticut and Massachusetts suggest a dynamic breeding range in the northeastern USA, likely a function of a warming climate [35] [36]. Due to this widespread and changing distribution within the species range, the large population size and the generally increasing populations [37], this species is of value as a model species. We are expecting this species to increase its range to the North (presumably due to climate change). Trends estimates for the red-bellied woodpecker were computed from time periods 1966–2009, 1966–1976, 1979–1983 and 1990–2002. These intervals were chosen to provide spatial trends over the period of study, and more specific time periods based on climatic variations hypothesized to be drivers of the red-bellied woodpecker dynamics.

## Results

### Spatial Approach vs Current BBS Analysis: How do the BCR Level Estimates Perform?

**Figure 1:**
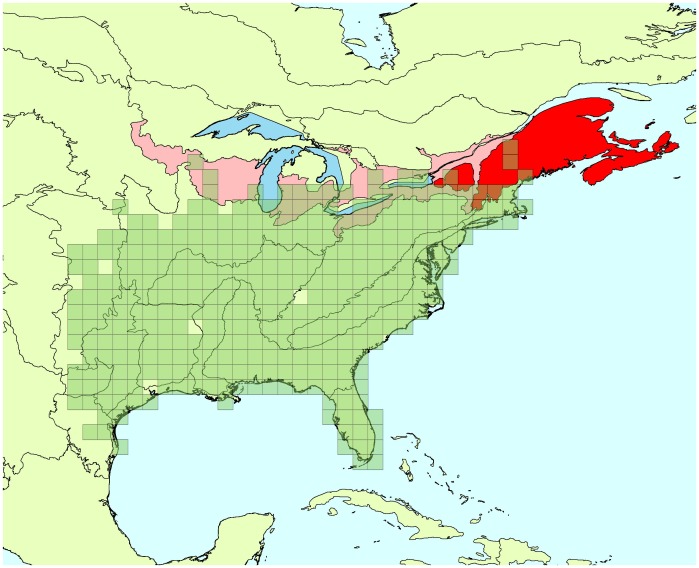
Carolina wren’s range and BCRs where trend estimates were improved by spatial approach. Green squares: cells where the species has been detected at least once between 1966 and 2009. Red areas: Bird Conservation Regions where trend estimates’ credible intervals were more precise due to the use of a spatial approach, compared to the current BBS analysis. Bright red indicates the BCRs where the improvement in precision is the greatest.

**Figure 2:**
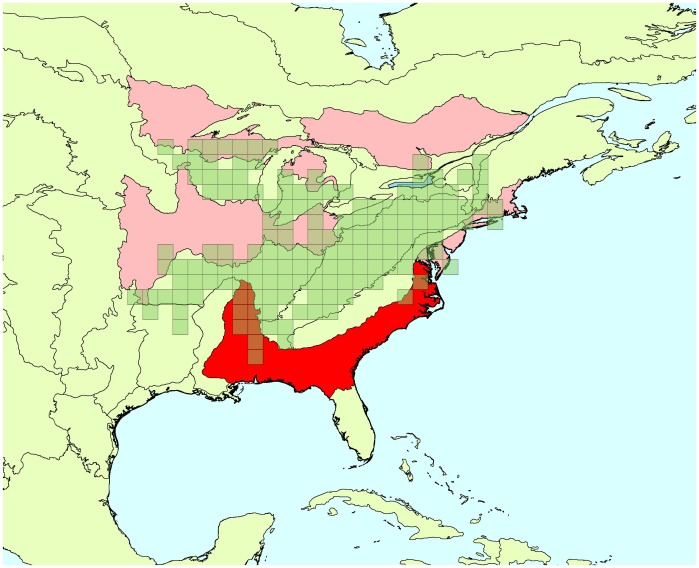
Cerulean warbler’s range and BCRs where trend estimates were improved by spatial approach. Green squares: cells where the species has been detected at least once between 1966 and 2009. Red areas: Bird Conservation Regions where trend estimates’ credible intervals were more precise due to the use of a spatial approach, compared to the current BBS analysis. Bright red indicates the BCRs where the improvement in precision is the greatest.

**Figure 3:**
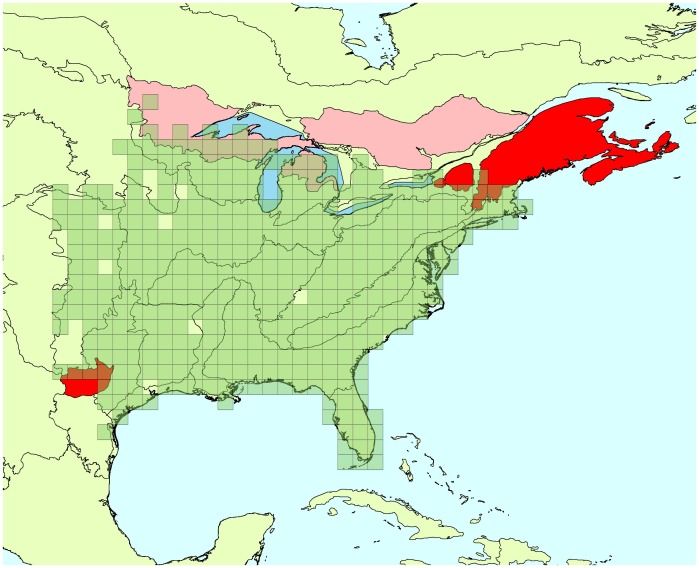
Red-bellied woodpecker’s range and BCRs where trend estimates were improved by spatial approach. Green squares: cells where the species has been detected at least once between 1966 and 2009. Red areas: Bird Conservation Regions where trend estimates’ credible intervals were more precise due to the use of a spatial approach, compared to the current BBS analysis. Bright red indicates the BCRs where the improvement in precision is the greatest.

Estimates of trend means are consistent between the two approaches (table 1). Approximately the same number of significant trend estimates (i.e., that have 95% CI that does not include 0) are present for the current BBS analyses (20) and the spatial analysis (17), over the 45 comparisons, and CI’s are smaller for the non-spatial analysis in 29 out of 45 comparisons (1 tie). However, when we look deeper into the estimates, we can see a difference between the two approaches in the width of the 95% CIs. Although, 95% CIs are on average 10% shorter with the spatial approach than with the non-spatial model, that shorter length is primarily a consequence of much smaller CIs in 1–2 regions for each species that are on the edges of the species ranges. Consequently, although the non-spatial estimates tend to be more precise in most regions, the lengths of the spatial model-based 95% CI tends to be more consistent: the global standard deviation for the mean length of the 95% CI is equal to 15.48 in the case of the spatial model and is equal to 26.22 for the current BBS analysis. This difference in precision in edge-of range BCRs conveys some benefits for analysis, as overall trends are influenced by extremely imprecise trends in edges of ranges. Average gain in precision for the Carolina wren is 10%, and this is driven by the Atlantic Northern Forest BCR (fig. 1). For the cerulean warbler gain is 11% on average, being driven by Southeastern Coastal Plain (fig. 2). The red-bellied woodpecker gain in precision is only 4% on average driven primarily by Atlantic Northern Forest and Edwards Plateau (fig. 3).

With a posterior spatial variance 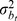 for the Gaussian CAR prior of 2.27 [2.17; 2.38], 4.76 [4.17; 5.26] and 1.72 [1.67; 1.85] respectively, the Carolina wren, cerulean warbler, and red-bellied woodpecker all exhibit spatial dependence. The magnitude of the conditional variance determines the amount of spatial variation. As spatial variance gets lower, the spatial dependence between neighboring spatial units becomes stronger, and therefore estimates in any given area tend to be more similar to that in neighboring areas [19] [38]. Spatial models have two aspects: strength of dependence and total amount of spatial dependence. In the Intrinsic CAR model, both aspects are controlled by a single parameter. A “small” conditional variance therefore indicates residual strongly dependent on neighboring values, and lead to a smoother general spatial structure [39].

### Carolina Wren

**Figure 4:**
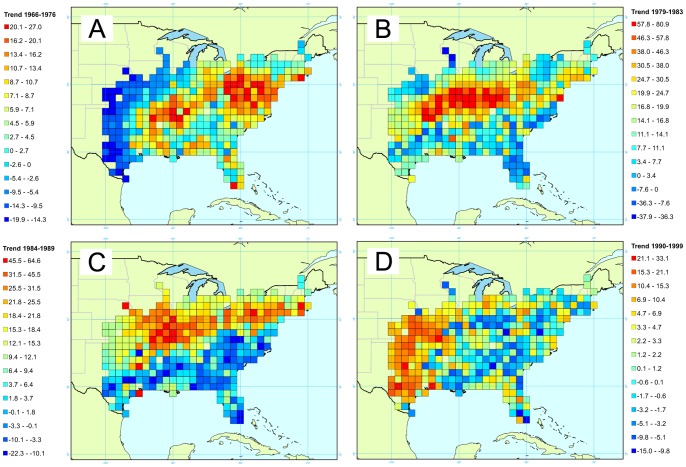
Map of the trends for the Carolina wren. Four times intervals are presented corresponding to yearly trends between A/1966 and 1976, B/1979 and 1983, C/1984 and 1989, and D/1990 and 1999. Trend is expressed as a percentage reflecting the yearly variation of abundance during the corresponding period.

Carolina wren data were collected over a total of 2,049 routes, distributed among 349 cells for a total of 449,054 detections, spread over 44 years and collected by 4,045 observers. Spatial patterns can be observed for the Carolina wren population trends, corresponding to four different time periods: from 1966 to 1976 (fig. 4A), from 1979 to 1983 (fig. 4B), from 1984 to 1989 (fig. 4C), from 1990 to 1999 (fig. 4D). During the first period –from 1966 to 1976– (fig. 4A), a decrease can be seen on the western part of the species range with decreases up to 20%/year while an increase in the central and northern parts of the species range is observed with trends as high as 30%/year. Two prominent areas of increase are in the center of the range and in the northeast. In the southern part of its range the Carolina wren population is stable. During the second period –from 1979 to 1983– (fig. 4B), most Carolina wren population decreases occurred in the southern portion of its range with Florida exhibiting the highest decrease (37%/year). During the same time period, increases as high as 80%/year occurred in the central portion of the range. The third time period –1984 to 1989– (fig. 4C) presents a distinction between the northern and southern portions of the range. In the south, the population decreased at a mean yearly rate of 15%, while the northern population increased by 20 to 65% per year. Finally, during the fourth time period –1990 to 1999– (fig. 4D), the Carolina wren populations in the western part of the range increased around 30%/year, while populations throughout the rest of their range experienced a moderate decreases (around 3%/year).

### Cerulean Warbler

**Figure 5:**
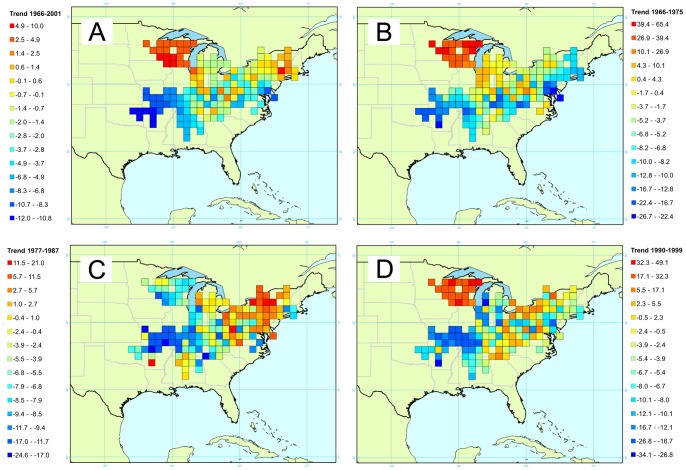
Map of the trends for the cerulean warbler. Four times intervals are presented corresponding to yearly trends between A/1966 and 2001, B/1966 and 1975, C/1977 and 1987, and D/1990 and 1999. Trend is expressed as a percentage reflecting the yearly variation of abundance during the corresponding period.

For the cerulean warbler, we have a total of 1101 routes, distributed among 154 cells for a total of 8,920 detections over a period of 44 years. These observations were collected by 2,269 individuals. We present four different maps: first, a global map corresponding to a period going from 1966 to 2001 (fig. 5A), then three maps demonstrating patterns for the following time periods: 1966 to 1975 (fig. 5B), 1977 to 1987 (fig. 5C) and 1990 to 1999 (fig. 5D). Three different distinct areas of population change can be identified in the cerulean warbler range, a northwestern spot, a southwestern spot, and a central eastern spot. Population in the northwest increased by 10%/year between 1966 and 2001, experiencing increases around 50%/year between 1966 and 1975, and between 1990 and 1999, while showing a decrease around 8%/year between 1977 and 1987. Populations in the southwest area showed a decrease of 10%/year between 1966 and 2001 (fig. 5A), with decreases up to 26%/year during the three time periods evoked. As for the central and eastern part, during the same time period, the population has been globally stable. This is particularly true from 1990 to 1999, while the population decreased slightly (around 5%/year) from 1966 to 1975, and increased slightly (around 10%/year) from 1977 to 1987.

### Red-bellied Woodpecker

**Figure 6:**
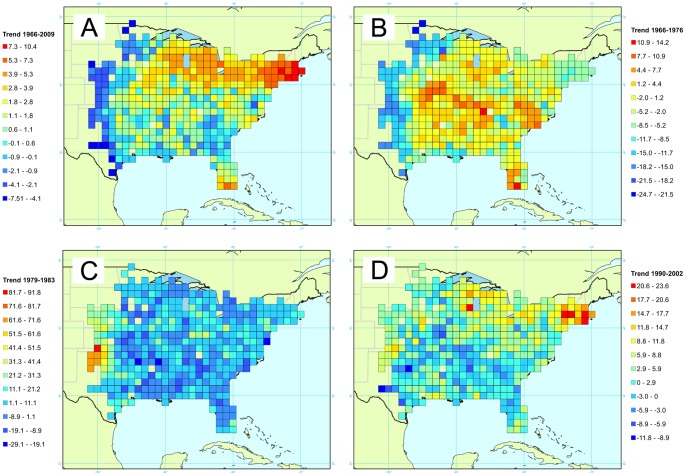
Map of the trends for the red-bellied woodpecker. Four times intervals are presented corresponding to yearly trends between A/1966 and 2009, B/1966 and 1976, C/1979 and 1983, and D/1990 and 2002. Trend is expressed as a percentage reflecting the yearly variation of abundance during the corresponding period.

For the red-bellied woodpecker, reports concern a total of 2,267 routes, over an area of 410 cells and represent a total of 314,381 detections, during a 44 years period. Those observations involved 4,549 observers. We present four maps for the red-bellied woodpecker. The first one shows the global trends from 1966 to 2009 (fig. 6A), the three subsequent ones offers a more time-detailed view for 1966–1976 (fig. 6B), 1979–1983 (fig. 6C) and 1990–2002 (fig. 6D) periods. The trend map over the whole time period shows a clear spatial pattern, with a steady population in the central area, and a high increasing front in the northern range, while at the most western limit of its distribution we observe a decrease (fig.6A). When we take a closer look at this pattern, we see that the acceleration of northern expansion occurred in the last 20 years. From 1966 to 1976, the species shows a global increase with yearly trends around 8–9%, while the western limit of the range presents negative trends around −15%/year (fig. 6B). For the time period 1979–1983 (fig. 6C) however, we have globally negative trends going as low as −30%/year. Finally, between 1990 and 2002 (fig. 6D) trends vary from negative trends in the south to positive trends in the northern part of the species range.

## Discussion

We have shown that incorporating a spatial component in the BBS analysis framework allows us to obtain relevant and biologically meaningful estimates for population trends. It also provides a flexible framework for obtaining trend estimates for any area, and with the beneficial effects of increasing precision in marginal BCRs relative to those estimated from the current approach used in the BBS analyses. Moreover, our model accurately reflects spatial sampling imbalance due to the manner in which BBS sample routes are allocated. The model effectively uses sample allocation units (degree blocks) as small geographic strata, and models spatial dependence among the units. We use a hierarchical model to model within-stratum variation and to model variation among strata in space and time.

A practical benefit of this model is that it tends to produce more precise estimates of trend in regions with very limited information. As the total amount of available data decreases, the importance of considering the spatial structure present in the dataset becomes more and more prominent to obtain more precise estimates. When available data are scarce, all supplementary information becomes relatively more important. Information arising from the spatial structure can be one of those. This could be the reason why in our analysis the increase in precision was specifically observed at the edge of species range. As the CAR model accommodates spatial dependence via a conditional specification, it allows for a “borrowing” of information across nearby units. This, thereby, effectively boosts the local sample size upon which trend estimation is based. In the complete absence of spatial dependence in abundance, then deriving stratum-based trend estimates from the underlying degree-block grid (and random effects model) should be basically equivalent to the non-spatial model based on “stratified random sampling” using a post-stratification scheme. Therefore, when abundance is correlated in space at a scale finer than the post-stratification scheme for which estimates are desired, we expect that the explicit spatial model should yield an improvement in precision in most cases.

A conceptual benefit of this model is that it allows producing estimates for arbitrary post-stratification schemes including even very small areas without having to rerun the analysis. This is because trend is a derived parameter from fundamental parameters that are defined by the design of the survey. As a result of this, there is complete consistency with respect to the model among any set of estimates based on different post-stratification schemes. Stratum-specific trends for different schemes are all simply reparameterizations of the basic underlying model parameters (see ‘Trend’ section above where we define a trend in terms of degree block quantities). Under our model, the grid-cell “effects” can be made available to the community and any arbitrary analysis (for different stratification schemes) based on those will retain consistency with the original analysis.

### Importance of Spatially Explicit Trends Models

In conservation biology, knowledge of the actual or potential distribution of a species is indispensable for threatened and endangered species management and protected area planning [40]. However, knowledge and a complete understanding of population trends appear to be equally essential and historically has been a key element for determining which species needs a particular management attention. This knowledge can only be achieved by a realistic approach of trends modeling that encompasses the complexity of spatial patterns.

More importantly, models that incorporate both spatial associations and associations of habitat and other factors influencing populations are critical for developing predictive models used in assessing the consequences of stressors such as habitat and climate change on bird populations. The models presented here achieve this goal, and should be useful in future modeling for management of bird populations.

Incorporating spatial autocorrelation in our model allowed us to obtain more precise results in regions where little information was available; this can be decisive in the management process. Surer information can help managers make more objective decisions. The use of spatial autocorrelation helps to improve the overall quality of trend estimates by explicitly accounting for underlying geographic variation in the data. In order to account for this spatial autocorrelation, and properly identify spatial patterns of trends, we used a CAR model. While the normal assumption for the CAR can be susceptible to outliers, potentially leading to local oversmoothing of avian counts [26] [41], Best *et* *al.* (1999) [41] found that the particular treatment of the spatial effects had little consequences on final model inferences, suggesting that the model framework is robust to such errors. We note that CAR models are widely-used for many applications of so-called “small-area estimation” [19] [20] [21] [22] in conventional survey situations such as arise in agricultural, epidemiology and census surveys. The conditional variance parameter 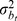 that controls the amount of variability in the spatial effect, and is *“a measure of the local variability conditional on the values of neighboring random effects”* [42]. It is common to incorporate both unstructured and spatially structured random effect to determine the importance of the spatial dependency. By determining if the spatially structured random effect is dominating the unstructured random effect, then estimates will display spatial structure. If it’s the opposite, the unstructured random effect will “*shrink the estimates towards the overall mean*” [25].

A benefit of including autocorrelation in a model is not only that the statistical assumptions are better met, but also that the predictive power of a model is improved by incorporating additional information or predictors, such as the values at neighboring locations [43] [44]. Bahn *et* *al.* (2006) [44] pointed out that not explicitly including spatial location in distribution models is based on the implicit assumption that species’ locations are independent in space and time. However, such an assumption could easily be violated if the conditions defining the species niche were autocorrelated; or if species’ locations were connected through dispersal or other behaviors that lead to spatial patterning such as aggregation or regular spacing. Interestingly, it should also be noted that spatial models can also improve variable selection [45] [46]. Non-spatial models cannot account for autocorrelation and thus may incorrectly select variables purely because they have a similar autocorrelation as the dependent variable and not because they are good predictors [45] [46] [47]. Therefore, the use of a spatially meaningful component in conjunction with other spatially varying covariates can help to determine exactly what part of a spatial pattern is due to the said covariates and what part is only due to spatial autocorrelation.

### Spatial Structures of Our Study Cases

The results obtained with our model coincide with what we know of the biology of the studied species, and allows us to obtain reliable maps and estimates of population trends.

Space-time population dynamics of the Carolina wren is a well-studied topic [48]. The observed trends in the most northern part of the species range correspond to the descriptions commonly applied to Carolina wren population dynamics. Explanations for those dynamics rely on several factors. The most evoked factor is the species sensitivity to cold weather: decimation of populations by severe winter conditions is well known [4] [49] [50]. The frequency of severe winters alternating with milder periods is the prominent reason for the constant regressions and expansions of the species Northern limit [51]. The effects of winter temperature, snow and ice on the Carolina wren abundance have been extensively studied [52] [53] [54] [55] [56]. For example, Bystrak (1979) [57], Graber and Graber (1979) [58] and Bohlen (1989) [55] have shown that populations size increases when winter temperatures are average but undergoes a significant drop during harsh winters (such as what happened between 1976 and 1977), possibly because in those periods food resources are not available, leading to starvation [4]. Those hypotheses are consistent with what we observed in this study. In our case, positive trends in the northern part of the species range during the periods 1979–1983 and 1984–1989 coincide with low winter precipitations. On the other hand, at the western part of the species’ range, the negative trends observed between 1966 and 1976 correspond to periods of low winter temperatures and high winter precipitations, while the positive trends between 1990 and 1999 match mild winters (Peter Blank, *pers. comm.*).

As expected, we saw that trends for the cerulean warbler greatly varied depending on the area with no specific regular pattern, and local variation of the population. For this species, it is known that conditions of both the breeding-season area and the winter habitat explain the changes in populations. While winter habitat could explain global variations of trends during the breeding season, local conditions appearing in the breeding season range are more likely to explain the observed spatial structure of trends during the time of the year data were collected. While the overall geographic limits of the breeding range have changed little during recent history, the relative abundance of the species within the range has experienced considerable change (mostly declined) since the early 1900s [59] [60]. This global decline in the species abundance and local variations of trends can be attributed to land-use changes emerging from increasing human populations in the breeding, migratory, and winter ranges [15] [59]. Humans have cleared habitats for other land uses and forest fragmentation is obvious in western and southwestern parts of the breeding range [61] [62]. It is also noted that the cerulean warbler has reoccupied areas when suitable habitat structure develops. Our model was able to detect those local changes in trends without oversmoothing the estimates which would have produced homogeneous maps.

We expected to detect an overall expansion of the red-bellied woodpecker population towards the northern part of its range, and our model was able to fully reflect that. While this species is a currently not a species of conservation concern, its dynamics still provide an interesting case study for trend modeling. Several factors have been presented in order to explain the different local patterns of spatial trends. Maturing forests in the Northeast and an increase in backyard bird feeders are thought to have contributed to the northern expansion of this species [63] [64]. Shackelford *et* *al.* (2000) [34] proposed that the range expansion towards the northwest was facilitated by following wooded river bottoms into the Great Plains where planted trees have matured in urban lots. Climate has also been cited as being responsible for some population declines, such as in Pennsylvania where the cold and wet breeding season of 1990 may have contributed to poor reproductive success [65]. Emlen *et* *al.* (1986) [66] have also concluded that the main explanatory variable for the red-bellied woodpecker range expansion was the latitude (while in the same study they failed to detect the effect of any environmental factors or latitude on the cerulean warbler and on the Carolina wren range expansions). By considering spatial autocorrelation in our model, we were able to take into account this effect of the purely spatial pattern (based on latitude), and an interesting element would be to add climate covariates to this model in order to determine which part of the trend structure is only due to the latitude and which part is due to climatic factors.

Among the common factors used to try to explain the bird population trends are habitat loss/changes, migratory status of the concerned species, climate/weather and other variables affecting reproduction and mortality (e.g., predation or parasitism, pollution).

Not surprisingly, most of the time, variation in abundance is the result of several different causes. Climate is an important variable that can affect species distribution and trends. DesGranges and Morneau (2010) [67] showed that for Quebec’s breeding birds after correcting for the effect of land cover variables, climatic variables explained 11.4% of the variation in the species distribution. Accounting for the effect of climate on population can be difficult because a very large portion of the variance that is explained by climate variables can be shared with spatial variables, reflecting the relationships among latitude, longitude, elevation, and climate [67]. However, identifying the direct effects of climate on bird distributions and the effects of habitat structure can be done by recognizing the distinctive distributional patterns of the two categories: smooth latitudinal (or altitudinal) gradients for climate, patchy mosaics for habitat structure [66]. Using our approach and by including climate covariates and possibly other spatially varying covariates, it would become possible to identify individually the parts of the species spatial pattern that are due to each covariates or simply to the spatial structure. The spatial random effect would indeed capture the spatial variation that is not related to other parameters.

Because all those variables can be responsible for variations in bird abundance, and because they can vary differently depending on the spatial location, continental trends may not reflect population trends at a more local level [68]. Consequently, as Peterjohn and Sauer (1994) [69] and Herkert (1995) [68] point out, managers should view the BBS data as indicators of the overall health of regional bird communities. A better understanding of current population trends and status is needed for managers to be most effective in conserving species.

On a statistical aspect, Thogmartin *et* *al.* (2004) [26] highlighted three challenges encountered when trying to model and map avian counts over space: extra-Poisson dispersion, nuisance effects associated with count data collection, and spatial autocorrelation. They noted that even if each of these challenges can be individually handled successfully with standard (i.e., frequentist) statistical approaches, there is currently no means for considering these challenges conjointly. We can also add that when modeling ecological dynamics on such a large scale on both time and space dimension, one issue that one will necessary encounter is the computational effort required to obtain estimates at a meaningful scale. When seen jointly with the even more important (and almost inevitable on such scales) issue of spatially imbalanced sampling, the accurate estimation of parameters and quantities such as trends becomes a juggling act. A trade-off between i) the spatial resolution of the grid, ii) the computation effort that can be invested in the study, and iii) the amount of data available for each stratum depending on the sampling design must be made in order to get ecologically meaningful and precise-enough estimates. Here, those issues were accounted for using a Bayesian approach. We were able to incorporate a spatial dimension into the existing framework used for trends assessment with BBS data. The next step is to provide a space model of bird population trends with covariates that could ultimately be used to provide predictive trend maps depending on expected climate changes. With such an approach, we will be able to help conservation efforts by determining how species will react to climate changes and ultimately to make BBS more useful to the conservation community.

## Supporting Information

Appendix S1 **WinBUGS code, including direct computation of BCR trend estimates.**
(DOCX)
