# Supplementary material for: Modeling Trends from North American Breeding Bird Survey Data: A Spatially Explicit Approach
Source: PLoS One. 2013 Dec 13;8(12):e81867. doi: 10.1371/journal.pone.0081867 (PMC3862490; doi:10.1371/journal.pone.0081867)
Supplement: Appendix S1 — WinBUGS code, including direct computation of BCR trend estimates. (DOCX) [file pone.0081867.s001.docx]

**Appendices**

| **Appendix 1.** WinBUGS code, including direct computation of BCR trend estimates. |
| --- |
| model  {  ##########################################################################  #  # ### Essential Data to provide ###  # nyear # Scalar, number of years in the study  # nroute # Scalar, number of sampled routes in the study  # ncell # Scalar, number of cells in the area of interest  # adjN[], weightsN[], numN[] # Neighborhood information for the CAR model  # N[r,t] # Matrix, detection data giving the total number of stops on which the species has been detected on route r at year t ; dim: nroute x nyear  # NbTotObs: # Scalar, total number of observers  # IDobs[r,t] # Matrix indicating the observer number (from 1 to NbTotObs+1) on route r at time t, IDobs[r,t]=NbTotObs+1 indicates that the route r has not been sampled during year t ; dim: nroute x nyear  # cell[r] # Vector indicating for each route the cell that contains they are in ; length: nroute  #  # ### Data to provide for the computation of BCR derived quantities ###  # nBCRinArea # Scalar, number or BCR appearing in the area of interest  # BCRweightinArea[c,BCR] # Matrix, giving for each cell the proportion of cell area contained in BCRs; dim: ncell x nBCRinArea  # yearA, yearB # Scalars respectively indicating the first and last years between which the trend has to be computed  # year1 # Scalar, the first year of the study, here =1966. Used to compute the column indices for yearA and yearB  #  # ### Parameters to initialize ###  # theta[t,1:ncell] # Spatial random effect at the cell scale, dim: nyear x ncell  # tauN # Scalar, precision for CAR model  # beta0[t] # Vector, year effect; length: nyear  # tauobs # Scalar, precision for observer random effect  # obs[l] # Vector ; observer random effect, length: NbTotObs+1  #  ###########################################################################  ### model ###  for (t in 1:nyear){  theta[t,1:ncell] ~ car.normal(adjN[],weightsN[],numN[],tauN)  beta0[t] ~ dnorm(0,0.1)  for (r in 1:nroute){  N[r,t] ~ dpois(muNtmp[r,t])  log(muNtmp[r,t]) <- beta0[t] + obstmp[r,t] + theta[t,cell[r]]    obstmp[r,t]<- longobs[IDobs[r,t]]  }  }  # observer effect  for (l in 1:NbTotObs){  obs[l] ~ dnorm(0,tauobs)  longobs[l]<-obs[l]  }  longobs[NbTotObs+1]<-obs[5] # Randomly chosen observer effect for years where a route has not been sampled  # precisions  tauobs <- 1/(sigmaobs*sigmaobs)  sigmaobs ~ dunif(0, 10)  tauN~dgamma(.1,.1)  ### derived quantities  # muN cells  for (t in 1: nyear) {  for( c in 1:ncell) {  log(muN[c,t]) <- beta0[t] + theta[t,c] # Computing the poisson mean for theoretical in cell c during year t  }  }  # muBCR  for (t in 1: nyear) {  for( BCR in 1:nBCRinArea) {  for( c in 1:ncell) {  muNBCRtmp[c,BCR,t] <- BCRweightinArea[c,BCR]*muN[c,t]  }  muNBCR[BCR,t] <- sum(muNBCRtmp[,BCR,t])/sum(BCRweightinArea[,BCR]) # Computing the average poisson mean for theoretical route in each BCR during year t  }  }  # trends BCR  indexB <- yearB-(year1-1)  indexA <- yearA-(year1-1)  for( BCR in 1:nBCRinArea) {  B_BCR[BCR] <- pow((muNBCR[BCR,indexB]/muNBCR[BCR,indexA]),(1/(yearB-yearA)))  trendBCR[BCR] <- 100*(B_BCR[BCR]-1) # estimating trend for each BCR between years A and B  }  } # end model |
